# Supplementary material for: Sub-molecular modulation of a 4f driven Kondo resonance by surface-induced asymmetry
Source: Nat Commun. 2016 Sep 26;7:12785. doi: 10.1038/ncomms12785 (PMC5052670; doi:10.1038/ncomms12785)
Supplement: Supplementary Information — Supplementary Figures 1- 5, Supplementary Discussion and Supplementary References [file ncomms12785-s1.pdf]

Supplementary Figures

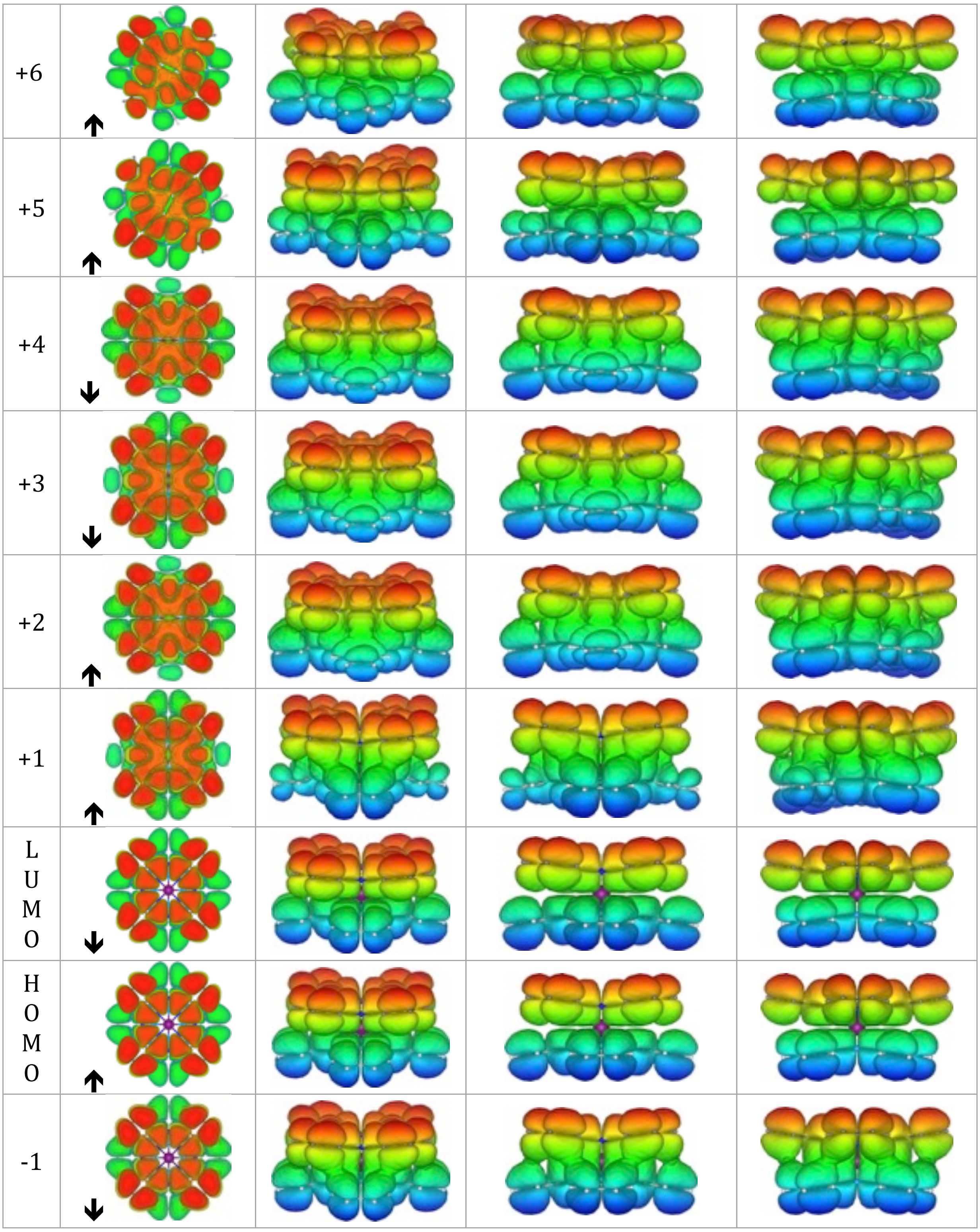

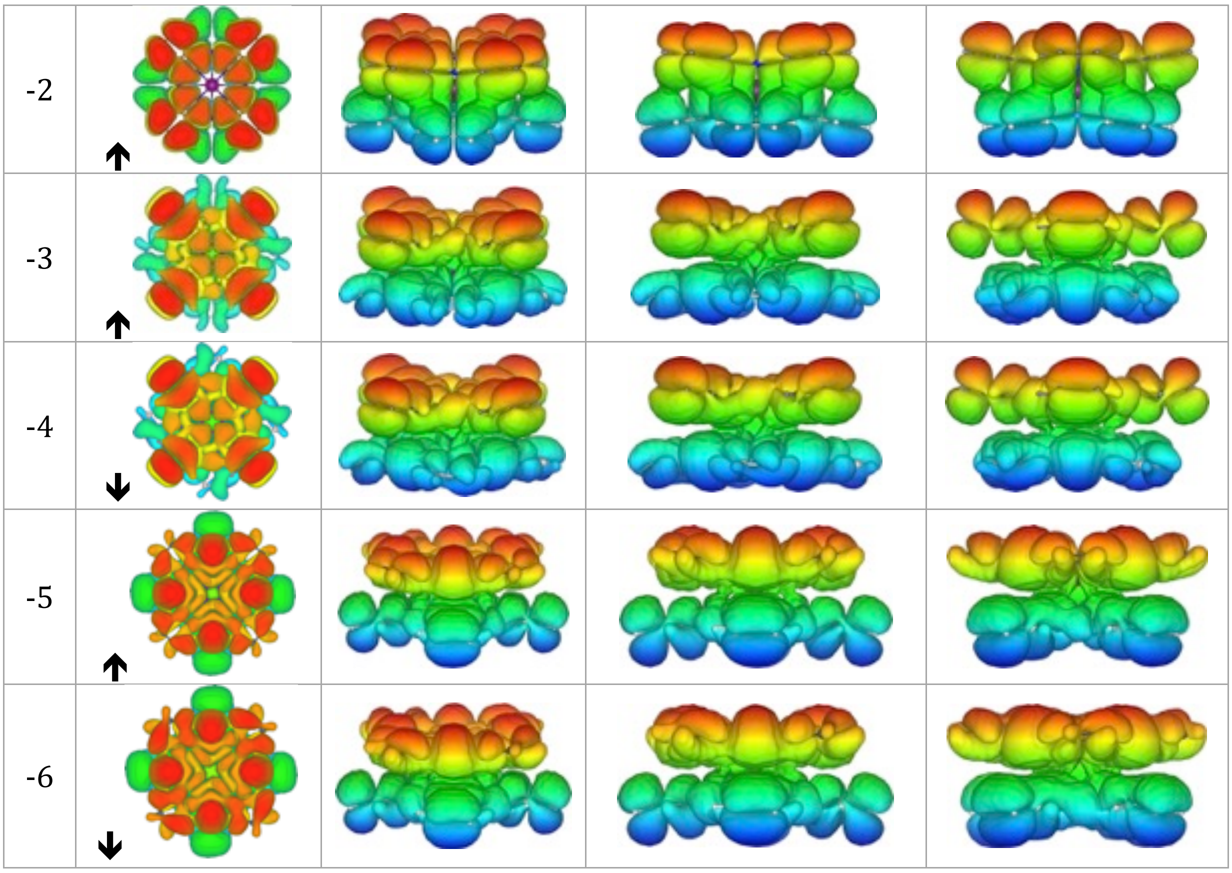

Supplementary Figure 1 – Charge density maps of molecular orbitals for isolated $DyPc_2$. Top and side views (with different tilt and rotation) of the charge density plots for selected occupied and unoccupied orbitals. The arrows indicate the spin channel (e.g. up or down) of the given orbital, and the colour scale specifies the height in 3D real space (blue is low and red is high).

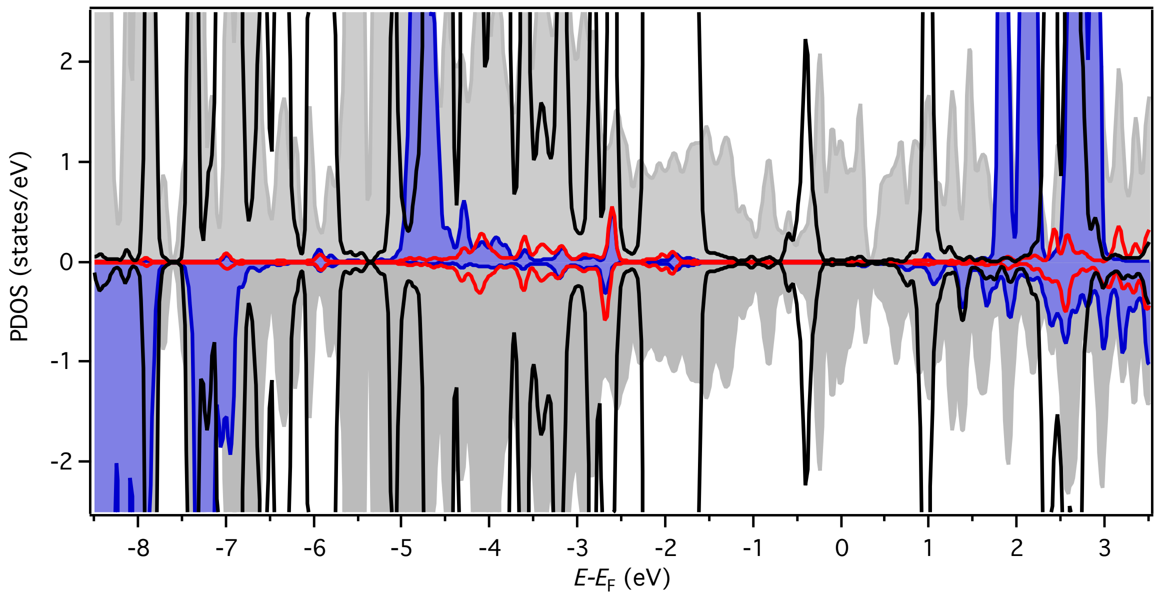

Supplementary Figure 2 – Spin-polarised PDOS of $DyPc_2$ on Cu(001). SP-PDOS of the adsorbed $DyPc_2$ molecule on Cu(001) substrate. For simplicity the PDOS of Dy atom shows only the projections onto the d- (bright red) and f- (purple) like atomic states while the lower (grey background) and upper (black) ligand sites show the sum of the projections onto the $s+p_x+p_y+p_z$ atomic orbitals of all Pc atoms.

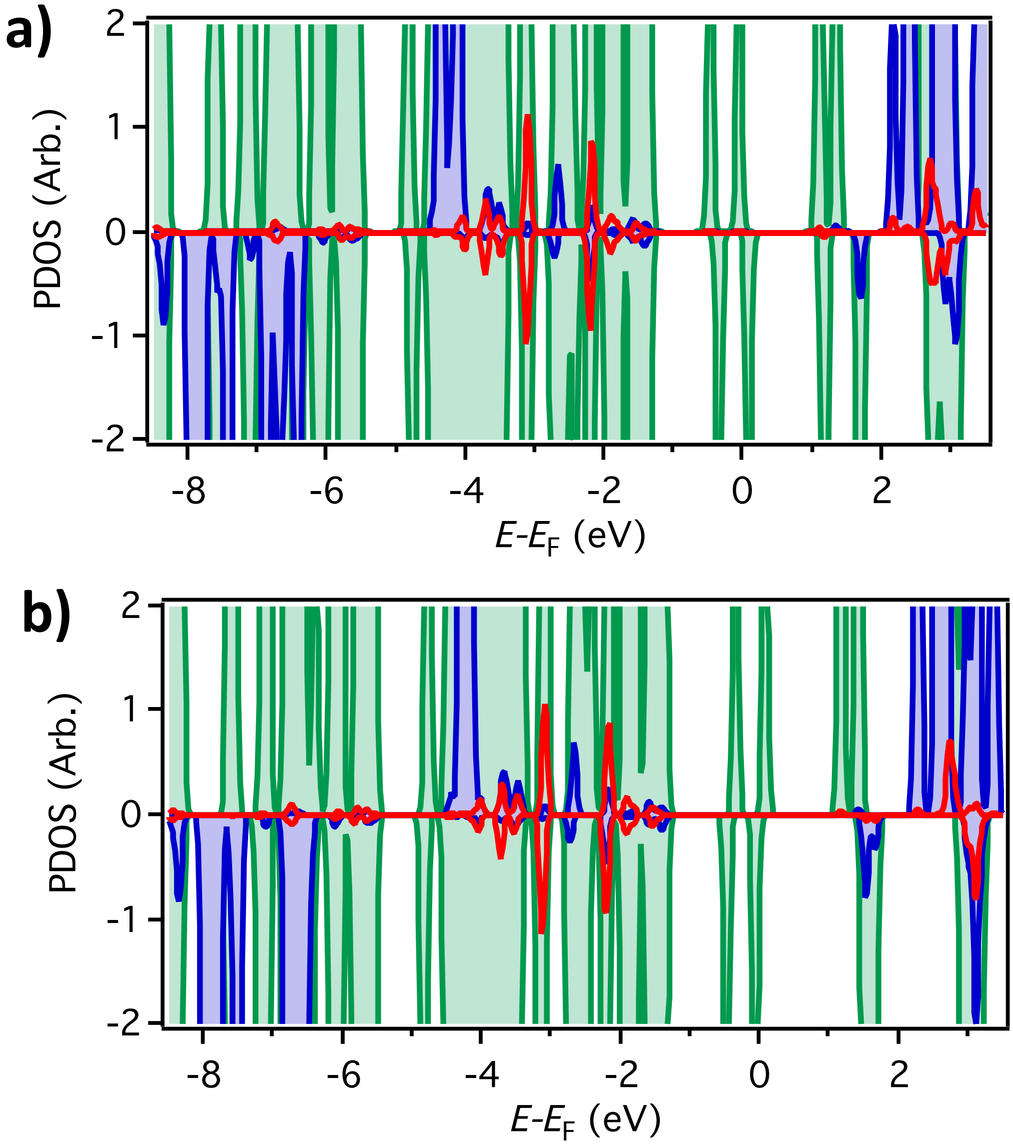

Supplementary Figure 3 – Spin-polarised PDOS for isolated $DyPc_2$. SP-PDOS for the AFM (a) and FM (b) configurations of the isolated (gas-phase) molecule. For simplicity the PDOS of Dy atom shows only the projections onto the d- (red) and f-like (purple) atomic states, while the ligand site (green line and background) shows the sum of the projections onto the $s+p_x+p_y+p_z$ atomic orbitals of all Pc atoms.

HOMO-1<br>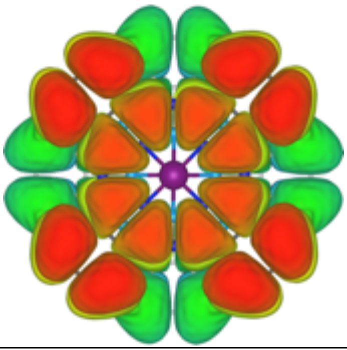	HOMO-1<br>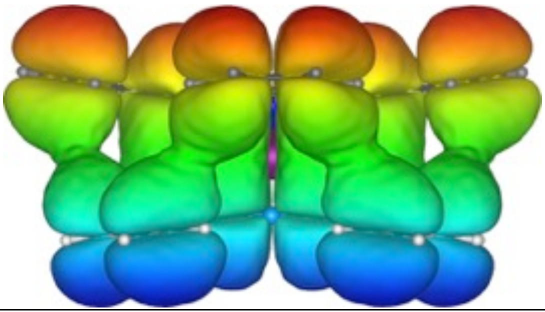
$DyPc_2$/Cu(001) substrate<br>STM topography for [-0.015 : E_F] eV<br>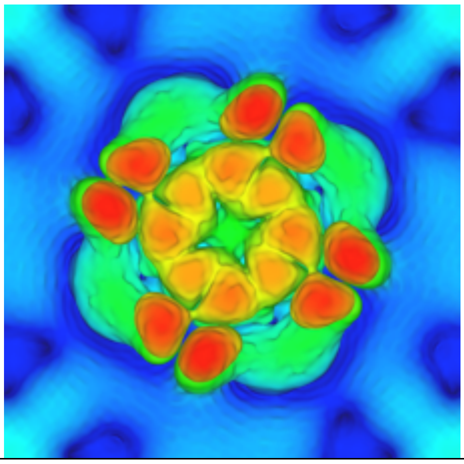	$DyPc_2$/Cu(001) substrate<br>STM topography for [-0.015 : E_F] eV<br>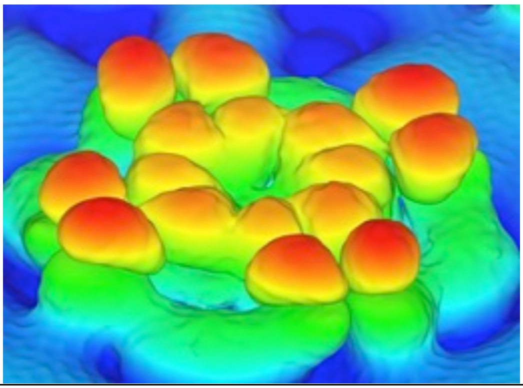
dI/dV map for +0.100 eV<br>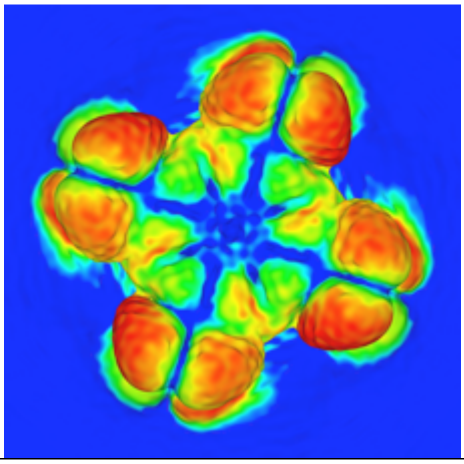	dI/dV map for +0.100 eV<br>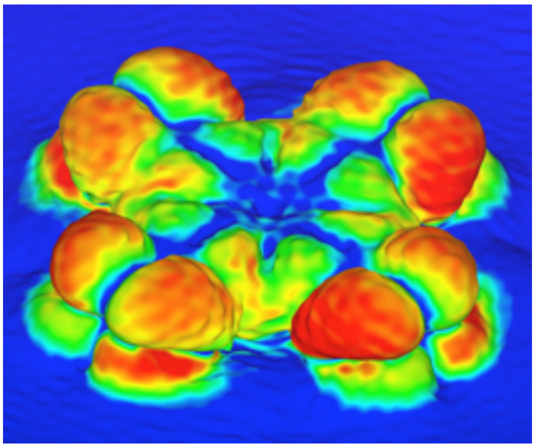

Supplementary Figure 4 – Charge density maps of molecular orbitals for $DyPc_2$ in isolation and on Cu(001). a) Top and side views of the charge density plots of an occupied orbital (HOMO-1) just below the HOMO for the isolated molecule. b) top and side views of the charge density plots found in a narrow energy interval just below the Fermi energy, e.g. [-0.015 : E_F] eV. The colour scale specifies the height in 3D real space: blue is low and red is high. c) Simulated top and side maps of the dI/dV in a narrow (±10meV) energy interval around 0.100 eV.

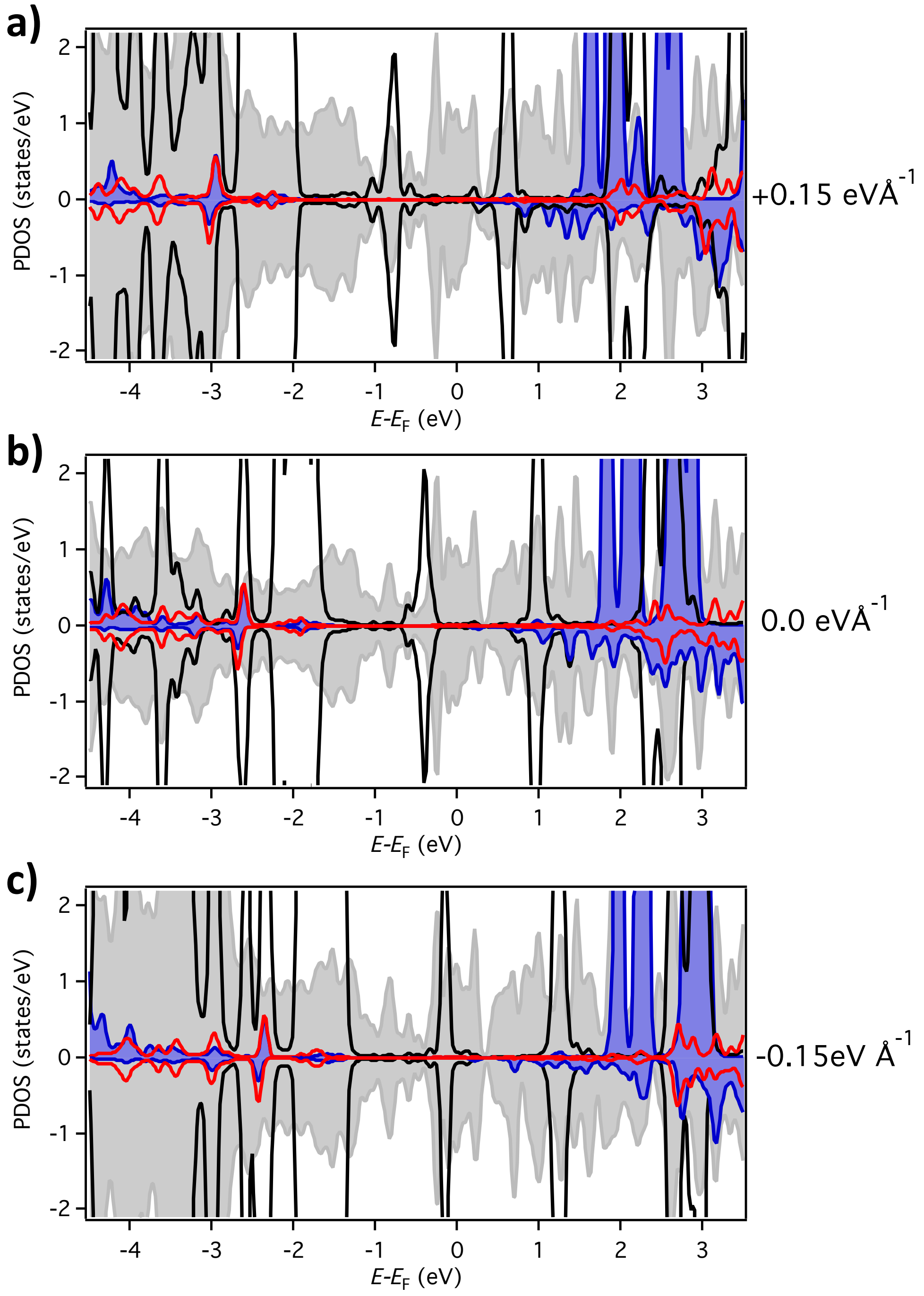

Supplementary Figure 5 – Influence of an external electric field. SP-PDOS for $DyPc_2$/Cu(001) system with and without an applied electric field, where positive (negative) electric fields are generated by negative (positive) bias voltages. For simplicity the PDOS of Dy atom shows only the projections onto the d- (bright red) and f- (purple) like atomic states while the lower (grey background) and upper (black) ligand sites show the sum of the projections onto the $s+p_x+p_y+p_z$ atomic orbitals of all Pc atoms.

Supplementary Discussion

Electronic and magnetic properties of the isolated (gas-phase) $DyPc_2$ molecule

An isolated Dy atom has a valence electronic configuration of $4f^{10}$ $5d^0$ $6s^2$, meaning that it has 12 valence electrons. In a simple (standard) ionic picture, only the $4f^{10}$ and $6s^2$ valence electrons in $DyPc_2$ are expected to interact with the Pc ligands, with the 2 6s electrons and 1 4f electron being transferred to and shared between the 2 Pc ligands. This would lead to 1 unpaired electron shared by the ligands, and therefore a 3+ formal oxidation state for Dy.

We calculated the ground state geometry and electronic structure of an isolated (single) gas-phase $DyPc_2$ molecule using an effective Hubbard U_{eff} of 6 eV. Our DFT calculations show that an on-site atomic hybridisation at the Dy site between the 4f and 5d is present. This leads to atomic-like hybrid orbitals with mixed df-character that hybridise with the Pc ligands. Within the DFT-suggested picture, the valence electronic configuration of Dy is hybrid-df^{10} $6s^2$. The projection of the total Dy charge density in an atomic sphere onto the atomic-like orbitals leads to the following quantities: 0.15 in s, 0.05 in p, 0.70 in d and 9.20 in f channels. Therefore, formally, for the Dy atom, (almost) 2 6s electrons are transferred to the Pc ligands and the central rare-earth ion can be considered as having a formal oxidation state of 2+.

The majority channel is represented by the spin down electrons while the minority channel is given by spin-up electrons. The total magnetic moment of the $DyPc_2$ for the AFM configuration (ground state) is 4 μ_B. This represents the difference between the 5 electrons found in the spin down at the Dy site and one unpaired electron in the spin-up channel, which is delocalized over the two Pc rings. As compared to the AFM ground state configurations, the FM configuration is higher by 9.11 meV and has a magnetic moment of 6 μ_B, which is the sum of the 5 electrons found in the spin down at the Dy site and one electron in the spin-down channel delocalized on the two Pc rings. Therefore, for the isolated molecule the magnetic exchange coupling between the Dy magnetic moment and the one carried by the Pc rings is evaluated within an effective Heisenberg Hamiltonian ($H = -2 \cdot J \cdot m_{Pc} \cdot m_{Dy}$) to J = 4.55 meV.

Although the f-states are localized at very negative bonding energies (effective Hubbard U_{eff} = 6 eV), the significant energetic difference between the AFM and FM magnetic configurations clearly indicates a strong interaction between the two magnetic moments, e.g. 5 μ_B of Dy and 1 μ_B of Pc ligands. In a simple picture, this suggests that the wave functions carrying these magnetic moments are overlapping to a quite significant extent in both spin channels. Assuming that the magnetic moment of the Dy atom is given only by the f-states, this large energy difference is an apparent contradiction because it is well accepted in literature that for late lanthanides the f-states are extremely localized in space and their overlap with the surrounding electronic states of other atoms is negligible (for example in 4f-metal oxides compounds).

However, the analysis of the calculated spin polarised density of states (SP-PDOS) of the $DyPc_2$ molecule reveals a scenario that originates from the specific chemical environment created by the (π electronic cloud of the) Pc ligands. More precisely, our simulations revealed that at the Dy ion an on-site atomic hybridization between the d- and f-atomic orbitals occurs. This leads to atomic hybrid states with mixed d- and f-character. Although in an isolated 4f-atom the f-states are very localized in space, the 5d states are much more delocalized in space and their tails extends further away from the atom. Therefore, an on-site atomic hybridization between the d- and f-states leads to atomic-like hybrid states at the Dy site with a significant f-atomic character and also extended in space that originates from the d-atomic like states. Due

to the long spatial extent, the tails of the Dy atomic hybrid df-orbitals can further significantly hybridize with the Pc ligands states. For example, these Dy-Pc hybrid states are clearly seen in the bonding states at -3.75, -3.10, -2.35 -1.90 eV and antibonding states at +1.5, +2.80 and +3.10 eV (see Supplementary Fig. 3).

Note that our analysis of the on-site hybridisation of the Dy-f states with the on-site s and d states for the isolated molecule (and also for the molecule on the surface) is based on a plane-wave code using the projector augmented wave method (PAW), which provides an all electron description for the valence electrons in a given atomic sphere. The radius of the atomic sphere is chosen during the generation of the PAW pseudopotential and kept fixed in all subsequent calculations. In particular, in our study we employed hard PAW pseudopotentials with atomic radii as small as possible to accurately describe the molecule-surface structural relaxation. Therefore, in the PAW formalism these small atomic radii set the lowest limit of the amount of charge density with s, p, d, f atomic-like character as compared to PAW pseudopotentials with larger atomic radii. The use of softer PAW pseudopotentials with larger atomic radii could only increase the electron counts found within the Dy atomic integration sphere and hence further reduce our estimate of the charge transfer to the ligands, compared to the hard PAW pseudopotentials employed in our calculations.

Ideally, in an isolated Dy atom the d-channel contains no electron. It is important to notice that when a Dy atom is placed between the two Pc ligands, as in the case of $DyPc_2$ molecule, the projection of the total charge density in a sphere around the Dy atom onto the s, p, d and f atomic like orbitals leads to the following quantities: 0.15 in s, 0.05 in p, 0.70 in d and 9.20 in f channels, respectively. These partial occupancies of the s, d and f channels strongly support our suggestion that in the $DyPc_2$ molecule the Dy atoms interacts with the Pc ligands via hybrid atomic like states that have mixed d and f character. However, the magnetization in each s, p, d and f atomic-like channels are: -0.004 (s) +0.011 (p) -0.026 (d) -5.024 (f). Therefore, we can conclude that (i) the electrons in the s, p and d channels are practically equally shared between the two spin up and down channels and (ii) the magnetic moment of the Dy atom originates from the 4f atomic-like orbitals. Furthermore, the total charge found in the sphere around the Dy atom (~10 electrons) implies that for the case of $DyPc_2$ molecule the formal oxidation state of Dy atom is 2+.

Electronic and magnetic properties of the $DyPc_2$ molecule on Cu(001)

Here we follow on from the discussions from the main paper: the adsorption of $DyPc_2$ onto the Cu(001) leads to a strong hybridization of the lower Pc ring with the surface and the ligand spin is quenched. The spin-polarised PDOS is shown in Supplementary Fig. 2. Importantly, the bonding between the Dy atom and the two Pc ligands for the $DyPc_2$/Cu(001) system is similar to the one of the isolated (gas-phase) $DyPc_2$ molecule.

In the case of adsorbed molecule, we found no net magnetic moment on the Pc ligands. Furthermore, the magnetization in each s, p, d and f atomic like channels is: -0.004 (s) +0.011 (p) -0.025 (d) -5.018 (f). The projection of the charge density in a sphere around the Dy atom onto the s, p, d and f atomic-like contributions leads to the following quantities: 0.16 in s, 0.07 in p, 0.72 in d and 9.21 in f channels, respectively. As in the case of the isolated molecule, we can consider that the Dy atom has a formal oxidation state of 2+ while the magnetic moment originates mostly from the 4f orbitals.

Details regarding the occupied and unoccupied orbitals for the isolated $DyPc_2$ molecule

Here we examine the charge density of selected occupied and unoccupied orbitals. As seen in Supplementary Fig. 1, we find that:

(i) HOMO and LUMO are π orbitals and have the same spatial distribution, with a nodal plane between the two Pc rings.
(ii) HOMO-1, -2 and LUMO+1, +2 are also π-like orbitals. Important to notice is that these π - orbitals are also delocalized between the two Pc rings (inner and outer part of the Pc ligands).
(iii) HOMO-3, -4, -5 -6 are molecular orbitals with π character on one Pc ring and a σ character on the other Pc ring.
(iv) LUMO+3, +4, +5, +6 have π character and are delocalized also between the inner part of the Pc ligands.

Supplementary Fig. 4a contains top and side views of the charge density plots of an occupied orbital (HOMO-1) just below the HOMO for the isolated molecule, while Supplementary Fig. 4b shows top and side views of the charge density plots found in a narrow energy interval just below the Fermi energy of the hybrid system, e.g. [-0.015 : E_F] eV. Although after $DyPc_2$ chemisoption new hybrid states between the $DyPc_2$ molecule and Cu(001) are formed, at the molecular site some of these states show common features with the molecular states also found in the isolated/single molecule. At the upper Pc ligand, the states in the energy interval [-0.015 : E_F] eV resemble more closely the HOMO-1 orbital of the single molecule that however is slightly rotated due to the adsorption of $DyPc_2$ onto the Cu(001) substrate. Intuitively, a simplified molecular interaction orbital picture is as follows: the chemisoption of $DyPc_2$ on the metal surface implies that the occupied molecular orbitals interact with the empty states of Cu(001) and the unoccupied molecular orbitals interact with the filled states of the substrate. As a consequence, bonding and antibonding combinations will be created. The HOMO-1 & metal states bonding combinations will appear at low bonding energy while its corresponding antibonding combinations will appear at higher bonding energies, in our case just below Fermi level. In Supplementary Fig. 4c, simulated top and side maps of the $\mathrm{d}I/\mathrm{d}V$ in a narrow (±10meV) energy interval around 0.100 eV are shown.

Electric field

As already demonstrated for similar systems [1], externally applied electric fields can energetically shift the states of the upper Pc ligand. As seen in Supplementary Fig. 5, if a positive uniformed electric field is added, the sharp electronic states (with a molecular-like character) of the upper ligand are shifted to lower energies, while for a negative uniform electric field these upper ligand orbitals are pushed to higher energies. Due to the metallic band-like character, the electronic states of the strong hybridized (lower) Pc ligand are practically not affected when including an electric field in the calculations.

Supplementary References

[1] Fahrendorf, S. *et al*. Accessing 4f-states in single-molecule spintronics. *Nature Commun.* **4**, 2425 (2013).
